# Evaluation of clinical interventions made by pharmacists, doctors and nurses in a hospital post-discharge telephone service: a retrospective cohort study

**DOI:** 10.1007/s11096-026-02207-y

**Published:** 2026-09-02

**Authors:** Kum Chuan Leong, Darshana Meanger, Philip Wu, Alex Wong, Wee Tjun Tee, Michelle Simango, Bianca Levkovich

**Affiliations:** 1 Eastern Health Pharmacy Department, Melbourne, VIC Australia; 2 Eastern Health, Melbourne, VIC Australia; 3 Monash University, Melbourne, VIC Australia

**Keywords:** Aged, Interventions, Medication-related problems, Nurses, Pharmacists, Physicians, Transition of care

## Abstract

**Introduction:**

Clinician-led post-discharge telephone follow-up programs are effective in resolving medication-related problems (MRPs) in older adults. Our hospital offers a post-discharge telephone service, Talk-To-You-Tomorrow (TTYT), to general medicine patients to support medication management following hospital discharge. However, little is known about the the types of interventions used to address MRPs between clinicians within post-discharge telephone services.

**Aim:**

To compare the types of interventions utalised (pharmacists versus non-pharmacist clinicians) in addressing MRPs for general medicine patients identified through a post-discharge telephone follow-up (TFU) service.

**Method:**

A retrospective cohort study investigated interventions provided to general medicine patients between June 2022 to May 2023 who underwent TTYT in a large metropolitan health service. Contemporaneous records were used to identify patients with MRPs and there corresponding interventions. MRPs were categorised using the Drug Evaluation Tools (NESA: necessity, effectiveness, safety and adherence). Interventions were classified using The Swiss Society of Public Health Administration and Hospital Pharmacists tools (patient-level, prescriber-level, drug-level and other interventions). Primary outcome was the difference in interventions (pharmacists versus non-pharmacist clinicians). Secondary outcome was the types of MRPs identified by different clinicians. Outcomes were evaluated using the Pearson’s χ^2^ test. Odds of MRP identification by clinician group were estimated with an adjusted odds ratio (OR) and 95% confidence interval (CI).

**Results:**

From 3,815 TTYT consultation records, 526 patients had MRPs. All MRPs had at least one intervention (227 pharmacist's and 299 non-pharmacist clinicians'). Pharmacists liaised with other clinicians the most (7.9% (18/227) vs 4.7% (14/299)) versus non-pharmacist clinicians who utilised more drug-level interventions (18% (53/299) vs 13.2% (30/227)). There were no statistically significant differences in the intervention types across pharmacists versus non-pharmacists (X^2^ (3, N = 526) = 4.271, p = 0.234) and MRP types identified by different clinicians (X^2^ (6, N = 526) = 11.777, p = 0.067). The odds of a pharmacist identifying an MRP was 2.27 times greater than non-pharmacists (p < 0.001, CI: 1.82–2.82).

**Conclusion:**

Clinicians mostly provided patient-level interventions however, no difference in the types of MRP’s identified in a post-discharge TTYT consult between various clinicians. Importantly, pharmacists were more likely identifying a MRP than other clinicians, which highlights the beneficial role of a pharmacist in medication management and their impact on medication safety.

**Supplementary Information:**

The online version contains supplementary material available at https://doi.org/10.1007/s11096-026-02207-y.

## Impact Statements

- Having a pharmacist in a post-discharge telephone follow-up service benefits patients by increasing detection of medication-related problems and resolving these in similar ways to other clinicians.
- The benefits to patients of a pharmacist in post-discharge follow-up services have been realised in the study service by increasing pharmacist staffing provision alongside service expansion.
- Dissemination of study results triggered the review of discharge processes and weekend clinical pharmacy service specifications. Extension in weekend clinical pharmacy services to facilitate discharge medication information provision is being explored.

## Introduction

Older patients and those with complex medication regimens face heightened risks of experiencing medication-related problems (MRPs) during the transition of care [1]. Particularly patients admitted to hospital under general medicine teams tend to be older and known to have complex admissions and medication regimens. Transition after prolonged hospital stay can result in a range of MRPs such as unintentional medication discrepancies, failure to re-start medications and inappropriate continuation of acute medications. These issues are aggravated by the lack of sufficient patient understanding on discharge instructions, leading to persistent symptoms and increased hospital readmission rates [1–3]. Ineffective communication among healthcare workers is a primary contributor to these challenges [2–5]. Various initiatives, including post-discharge telephone follow-up (TFU) programs, have been implemented to improve patient understanding of discharge instructions, mitigate medication misadventures, and enhance overall health outcomes [1, 4, 6–8]. Systematic reviews underscore the effectiveness of post-discharge TFU interventions in reducing all-cause admissions, emergency department visits, and mortality rates across diverse patient cohorts [9, 10].

Recent studies have described the spectrum of interventions utilised in managing MRPs identified during TFU [11–13]. These include correction of medication discrepancies, patient education and optimisation of therapies However, there is limited literature systematically comparing and assessing the effectiveness of these interventions in addressing MRPs, particularly how they are provided by various healthcare professionals such as pharmacists, physicians, and nurses, each possessing distinct expertise and training [14]. Interventions targeting MRPs could be heterogeneous, contingent upon the clinician involved and the specific nature of the issue. The Pharmaceutical Care Network Europe (PCNE) classification framework serves as a prevalent taxonomy, categorising interventions into four primary domains: patient-level (e.g., educational initiatives, compliance enhancement), drug-level (e.g., dosage adjustments, therapy modifications), prescriber-level (e.g., clinical oversight, therapy monitoring), and other interventions (e.g., healthcare provider coordination and liaison) [15]. This complexity in nature of intervention strategies highlights the importance to understand which intervention types are used by different clinicians in mitigating MRPs.

Pharmacist-led telephone consultations studies and outpatient clinic review often address MRPs through patient-level interventions and dose adjustments or regimen modification by collaborating with patients' regular physicians [4–6, 16–18]. Post discharge pharmacist reviews have highlighted the burden of MRPs such as unclear indications and non-adherence and have been shown to reduce readmission rates [16–18]. Previous studies have shown either cost-effectiveness or reductions in hospital readmissions secondary to MRPs but have not differentiated interventions used across healthcare disciplines potentially due to the lack of interdisciplinary research [4–6]. Concurrently, medically led interventions in cardiology and endocrinology have resolved MRPs by initiating new medications or adjusting drug regimens [11, 19]. Additionally, outpatient diabetes nursing-led telephone consultations effectively identify diabetes-related MRPs, offering education and monitoring via telecommunications [20]. While these studies highlight the significance of MRPs and interventions, there is limited discussion on variations in the types of interventions utilised among different clinicians.

## Aim

This study aimed to compare the types of interventions utilised by pharmacists as compared to non-pharmacist clinicians in addressing MRPs for general medicine patients that were identified through a post-discharge TFU service. A better understanding of the types and differences in clinicians' interventions will support ongoing service delivery optimisation.

## Method

### Study design and setting

This was a retrospective cohort study. The STROBE criteria for reporting of cohort studies have guided the design and reporting of the study [18].

**Fig. 1:**
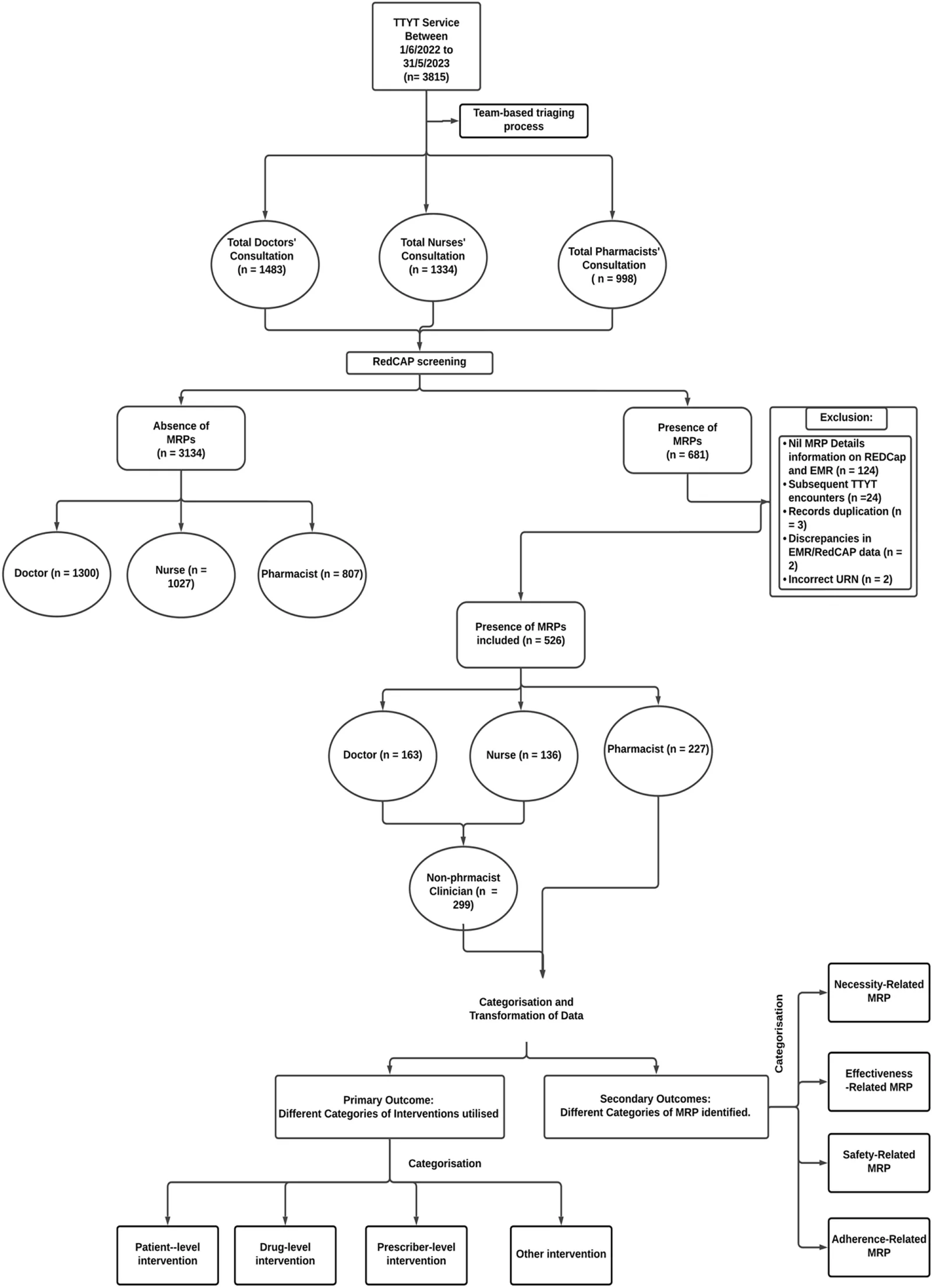
Study flow chart of participant identification, data handling and analysis. *MRPs* medication related problems, *TTYT* Talk-to-you-tomorrow service, *EMR* electronic medical records, *URN* unique record numbers, *REDCap* Research Electronic Data Capture.

The study was conducted in the general medicine unit at Eastern Health, a large tertiary public metropolitan health service in Melbourne, Australia. Annually, approximately 9,500 patients were discharged from the general medicine unit. As part of routine practice, eligible patients were triaged for TFU through the Talk To You Tomorrow (TTYT) service at the point of discharge (Fig. 1).

The TTYT clinic was staffed by a multi-disciplinary team. The TTYT medical clinician in collaboration with TTYT clinic staff would triage patients to each clinician based on their needs (e.g. patients with multiple medication changes would be allocated to the pharmacist). Patients that had been recently discharged form a General Medicine ward were triaged at the beginning of the shift. TTYT clinicians included general medicine consultants or geriatricians, registered nurses with a background in geriatrics or general medicine, and clinical pharmacists with experience in general medicine. TFU occurred within 24–48 h post-discharge. Each 20–30-min telephone call addressed general well-being, discharge paperwork, medication review, and referrals for ongoing care. Clinicians ensured medication understanding, addressed MRPs, and, if required, necessary referrals were made. Consultation records were made in the electronic medical record (EMR) as well as a specific TTYT service database. TTYT consultations were included in the study based on the following:

### Participants

Inclusion criteria:

- Adults' patients aged 18 years or older, discharged from the Eastern Health’s General Medicine Unit between 1st June 2022 to 31st May 2023
- Completed TTYT telephone service.
- MRPs identified during their initial TTYT consultation during the study period and recorded data in both Research Electronic Data (REDCap) and EMR.

Exclusion criteria:

- Subsequent TTYT consultations for the same patient during the study period.

### Variables

#### Outcomes

The primary outcome was the difference in intervention categories utilised by pharmacists compared to non-pharmacist clinicians in addressing the MRPs identified during the TTYT consultations. Secondary outcomes were the difference in MRP types, the potential factors that contribute to patients’ MRPs and the medications involved in the identified MRPs.

### Data sources, collection and handling

Data was extracted from routine care and TTYT consultation records using REDCap [19].

Demographic information for all patients who had TTYT consults was extracted from REDCap and eligibility criteria applied (Table 1). Additional patient information for patients who had at least one MRP identified was manually collected from the EMR and local medication dispensing software, Merlin®.

**Table 1:** Patient Characteristics of patients with medication related problems (MRP) including comparison with patients without MRPs

	Patients with MRPs identified (n = 526)	Patients without MRP identified (n = 3134)	p-value
Male gender, n (%)	256 (48.7)	1469 (46.9)	0.693
Age, Median years (IQR)	81 (75–87)	81 (75–87)	0.998
Median Length of stay (days) (IQR)	4 (2.8–7.0)	4 (2.0–7.0)	0.878
Primary Language			
English	457 (86.9)		
Median Age-adjusted Charleson Comorbidity Index (IQR)	5 (4–6)		
Medication Management			
Self	429 (81.6)		
Carer/Family members	79 (15)		
Facility Staff/ Nurse	17 (3.2)		
Administration Aid, (%)			
No	345 (65.7)		
Altered dose form, (%)			
No	516 (98.1)		
Number of medications before index admission per patient, Median (IQR)	10 (7–14)		
Total Changes in medication as compared to home medications on discharge (New/Change/Stop)	512		
Median changes (IQR) per patient	4 (2–6)		
Number of medications prescribed on discharge	5928		
Median (IQR)	11 (8–14)		

n = Number; MRPs = Medication related problems IQR = Interquartile Range; % = percentage

Data collection was conducted by the principal investigator (KC), who occasionally participated in TTYT service, and data curators (WT and MS) who were not directly involved in the TTYT service provision. TTYT consultations involving the principal investigator were allocated to other data curators for data collection to mitigate any potential information bias. Any coding disagreements were discussed with data curators and if a consensus could not be achieved a third coder was introduced. Coding book was updated with the final consensuses.

### Statistical analysis

The one-year sample of convenience was selected to capture seasonal medical condition variation. The resulting sample size was similar to, or larger than, adequately powered studies [12, 13]. Data analysis focused on the primary MRP identified for a patient and its corresponding intervention to maintain data independence. Descriptive statistics were used to compare number of intervention types and MRPs identified across clinician groups, and no formal statistical testing was performed.

Statistical analysis with statistician input was conducted using SPSS 19.0 (SPSS Inc., Chicago, IL, USA) and Microsoft Excel®. Pearson’s χ^2^ test assessed the relationship between intervention types or MRP types and clinician types, with a p-value < 0.05 considered statistically significant. Baseline patients' characteristics were compared using the χ^2^ test for categorical data and Mann–Whitney U for continuous data. Comparisons between MRPS versus non-MRPS were only made for age, length of stay and sex due the large number of non-MRPs and limited data collection resourcing. Binary logistic regression examined MRP detection rates among clinicians and potential influencing factors like age, length of stay, gender, and interpreter requirement. Significant results were reported with adjusted odds ratios (OR) and confidence intervals.

MRPs were transformed into categorical data using the Drug Evaluation Tools (NESA: necessity, effectiveness, safety and adherence) developed by Monash University, informed by University of Alberta transition tool framework (Fig. 1 and Supplementary Information SI-1) [20, 21]. Interventions for identified MRPs were classified using tools from The Swiss Society of Public Health Administration and Hospital Pharmacists (GSASA) which uses the four domains of the PCNE framework (patient-level, prescriber-level, drug-level and other interventions) (Fig. 1) [15, 25]. Data transformations were guided by a validated codebook [26].

Medications implicated in the identified MRPs were categorised utilising the Anatomical Therapeutic Chemical (ATC) classification system [27]. Their associated risk levels were determined and categorised using the High-Alert Medications Stratified Revised (HAMST-R) tool [28]. Discharge summaries and admissions notes were utilised to calculate patients’ Age-adjusted Charleson Comorbidities indexes (a-CCI) [29].

### Ethics approval

This study has undergone review and approval by the Eastern Health Human Research Ethics Committee (LR23-055–100928) and Monash University (40,142) on 27th September 2023.

## Results

During the study period 3,815 TTYT consultations were recorded; 681 patients had at least one MRP identified, of which 526 met eligibility criteria and were included (Fig. 1). Among the total sample, the average age was 81, and 48.7% were male. There were no statistically significant differences detected in the age group, gender and length of stay in patients with or without MRPs identified (Table 1).

Amongst the patients with MRPs identified, the median a-CCI for patients was 5 (IQR4-6) indicating a high burden of comorbid conditions (Table 1). Patients experienced a median of 4 (IQR 2–6) changes of medications during the index hospital admission and had a median of 11(IQR 8–14) medications prescribed on discharge.

A total of 526 patients categorised MRP-related interventions were analysed. Pharmacists made 227 interventions, and 299 interventions were made by non-pharmacist clinicians (Fig. 1).

### Primary outcome

**Fig. 2:**
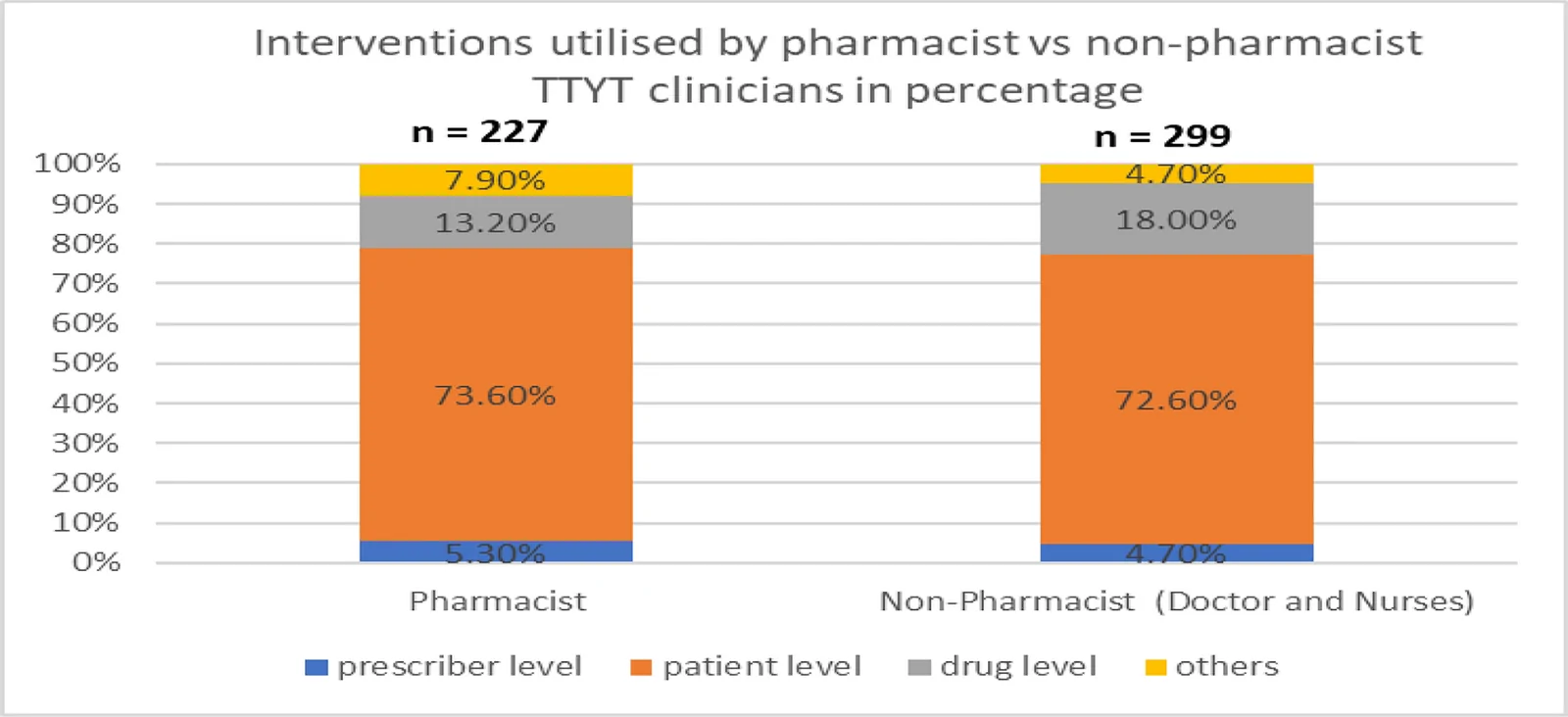
Interventions utilised by pharmacist versus non-pharmacist clinicians classified as prescriber-level interventions, patient-level interventions, drug-level interventions and other interventions

There was no statistically significant difference in the types of interventions (patient-level, drug-level, prescriber-level or other) utilised between pharmacists and non-pharmacist clinicians (X^2^ (3, N = 526) = 4.271, p = 0.234) (Fig. 2). However, statistically significant differences were observed when pharmacists (X^2^ (3, N = 390) = 10.9, p = 0.012) or nurses (X^2^ (3, N = 299) ≥ 16.27, p < 0.001) were individually compared to doctors, but no difference was found when comparing pharmacists to nurses directly (X^2^ (3, N = 363) = 5.5, p = 0.135).

Patient-level interventions emerged as the most frequently employed by both groups (Fig. 2). The least utilised prescriber-level interventions were also similar in both pharmacists and non-pharmacist groups (5.3% (12/227) vs 4.7% (14/299). Notably, non-pharmacist clinicians exhibited a higher proportion of drug-level interventions compared to pharmacists (18% (53/299) vs 13.2% (30/227)). Pharmacists utilised more interventions such as liaison with other clinicians in contrast to non-pharmacist clinicians (7.9% (18/227) vs 4.7% (14/299)).

### Secondary outcomes

Among all TTYT records, medical consultations constituted the largest share. They exhibited a comparable percentage of MRP identifications (10.9% (163/1483)) to those identified by nursing staff (10.1% (136/1334)) but a lower percentage compared to pharmacy (22.7% (27/998)) (Fig. 1). The odds of a pharmacist identifying an MRP were 2.27 times greater than a nurse or doctor (adjusted OR 2.267; p < 0.001, CI [1.82–2.82]).

**Fig. 3:**
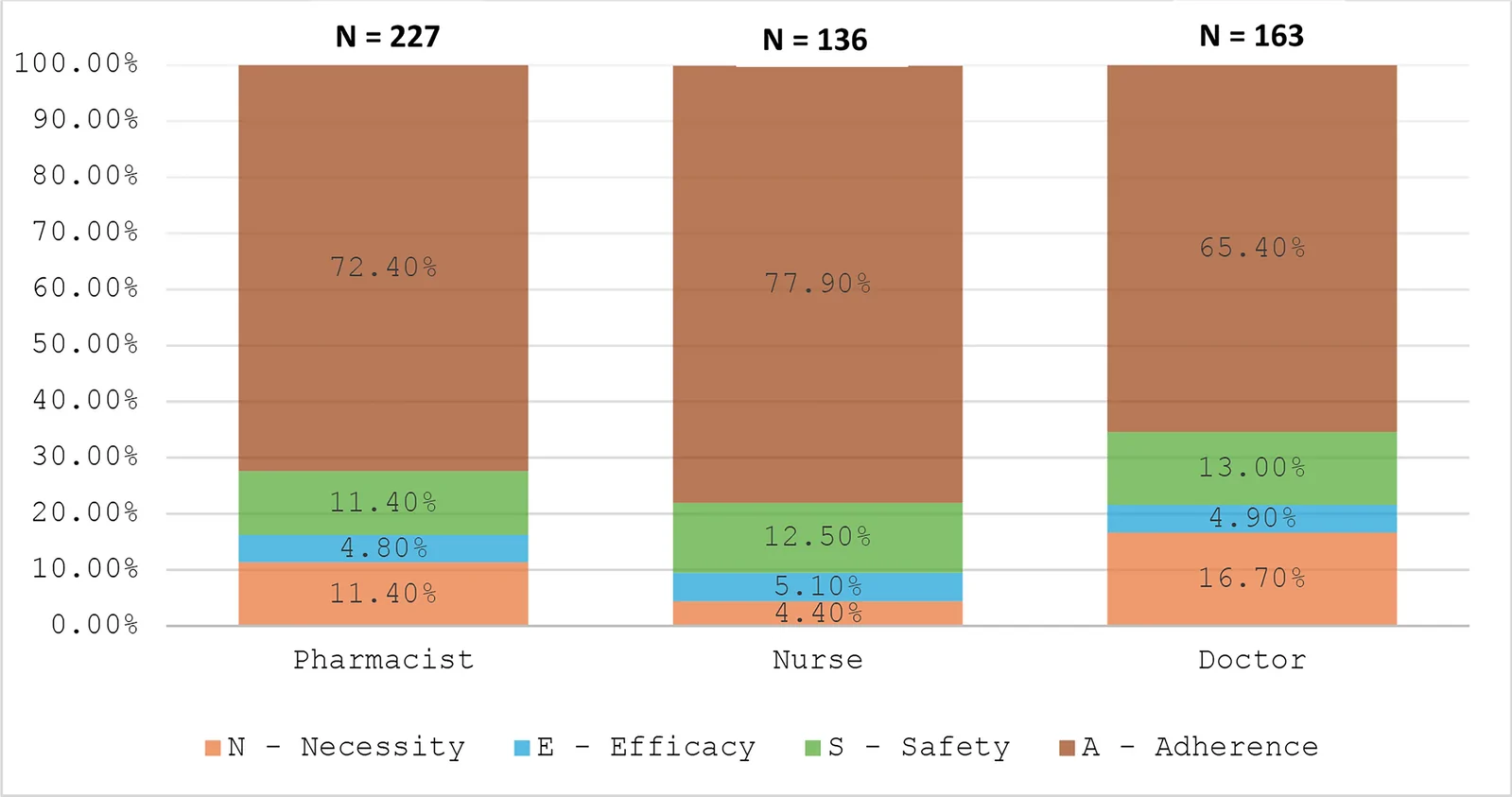
Different types of Medication Related Problems identified by different clinicians categorised using NESA tool

Despite differences in the MRP detection rates amongst clinicians, there was no difference in the MRP types identified (X^2^ (6, N = 526) = 11.777, p = 0.067) (Fig. 3). Of the 526 MRPs identified adherence-related MRPs emerged as the most frequently identified (Fig. 3). Doctors and pharmacists identified significantly more necessity-related MRPs as compared to nurses. Meanwhile, the efficacy and safety-related MRP rates were relatively consistent across all clinicians.

Adherence-related MRPs were mostly resolved with patient-level intervention, which was consistent across the board, with nursing at 88.7% (94/106), pharmacy at 81.1% (133/164), and medical at 74.5% (79/106). The distribution of different types of interventions employed by different clinicians to resolve specific MRPs is summarised in Supplementary Information (SI-4) with some illustrative examples (Supplementary Information SI-2).

A total of 805 medications were associated with the identified MRPs (Table 2). Most identified medications belonged to cardiovascular system (31.6% (254/805)), alimentary tract and metabolism medications (18.5% (149/805)), nervous system medications (15.2% (122/805), and anti-infective agents for systemic use (9.4% (76/805)) (Supplementary Information, SI-3 and SI-5). With HAMST-R scores of 4 or greater but less than 7, 13.8% (111/805) of the medications were classified as high-alert medications. The remainder were classified as low-alert as no medications posed the highest risk (HAMST-R > 7).

**Table 2:** Medications identified as contributing to medication related problems classified according to WHO ATC groupings

Medications grouped by ATC groupings	Number (%) N = 805
A: Alimentary Tract and Metabolism	149 (18.5%)
B: Blood and blood-forming organs	83 (10.3%)
C: Cardiovascular system	254 (31.5%)
D: Dermatologicals	3 (< 1%)
G: Genito urinary system and sex hormones	2 (< 1%)
H: Systemic Hormonal preparations, excluding sex hormones and insulins	26 (3.2%)
J: Anti-infective for systemic use	76 (9.4%)
L: Antineoplastic and immunomodulating agents	2 (< 1%)
M: Musculoskeletal system	23 (2.9%)
N: Nervous system	122 (15.1%)
P: Antiparasitic products, insecticides and repellents	1 (< 1%)
R: Respiratory system	54 (6.7%)
S: sensory organs	10 (1.2%)

*The number of Medications is greater than the number of MRPs because one MRP would sometimes have more than one medication. From our study, 19 MRPs involve four medications, 41 MRPs involve three medications, 141 MRPs involve two medications and 324 MRPs involve one medication

None of the variables analysed demonstrated significant predictive power for the presence of MRPs with p > 0.05 for all potential variables and low Nagelkerke R^2^ value (0.034).

## Discussion

This study reviewed clinician interventions across 3,815 general medicine patients receiving TFU at a major metropolitan hospital. The results demonstrated that pharmacists and other clinicians use similar interventions to address MRPs identified through the TTYT service. Clinicians primarily provided adherence-related interventions through the TTYT service (e.g. non-adherence and over-adherence). No difference was observed across the types of MRPs identified by different clinicians.

The increasing emergence of TFU services is beneficial for patients during their transition of care and reduce re-admission rates [5, 7, 8, 16–18]. To our knowledge, this study is among the first to compare the approach of different clinicians to resolving MRPs within these services. When comparing individual disciplines, medical staff employed different types of interventions compared to pharmacists or nurses. This observation is expected, as doctors were the only prescribers within the TTYT service team. This finding may be different in settings where pharmacists or nurses are also prescribers.

Overall, patient-level interventions were the most frequently employed by both pharmacists and non-pharmacist clinicians (e.g. patient counselling). These findings align with existing literature exploring pharmacist interventions in post-discharge services in face-to-face clinic settings, including de Gregori et al. (69.5%) and Tong E et al. (65%) [30]. Further, Ibrahim NA et al. demonstrated that physicians in geriatric clinics employed patient-level interventions at a rate of 54%, which is comparable to the 63% observed among physicians in our TTYT service [31, 32]. The slightly higher rates observed in our study may be influenced by the remote nature of the consultation by telephone [30]. However, in a telephone consult service Hu et al. reported 34% usage of patient-level interventions post-discharge by nurses, contrasting our findings, likely due to the specific focus on diabetes regimen titration in that study [20].

Our study found that pharmacist were more likely to liaise with other clinicians during their consultants compared to was non-pharmacist clinicians. Notably, most interventions by non-pharmacist clinicians, as reported in the available literature, did not reveal liaison with other health practitioners as part of their study design. In contrast, pharmacist-led services have incorporated multi-disciplinary liaison and the utilisation of other interventions [32, 34]. Although this complicates direct comparisons of other interventions between clinicians, it underscores the collaborative approach of pharmacists to patient care particularly MRP resolution.

We found pharmacists were more likely to identify a MRP compared to other clinicians. While this finding is intuitive given pharmacists focus and skills in assessing medicines use and safety, direct comparison with other studies is difficult as there is limited evidence available. The MRP identification rate in our study represents approximately 18% of all TTYT consults. A systematic review of transitional care medication-related harm indicated a higher rate of 54% [35]. This contrast is unsurprising as this review included studies that reported medication errors and medication-related harm in all types of follow-up services. Our study reviewed patients within 24–48 h of discharge, while other studies followed patients for an average of one month but some up to three months post-discharge.

## Limitations

There were several limitations in this study. Variability in staffing at TTYT, influenced by rostering and leave schedules, introduced confounding factors due to differences in staff experience, training, and educational levels. The absence of a standardised diagnostic tool for MRPs further complicated the consistency of MRP identification and intervention, potentially impacting study outcomes. Moreover, manual data entry during consultations introduced variability and potential errors related to individual practices, documentation bias and technology proficiency, affecting the thoroughness of data capture. However, these factors contributed to the findings reflecting the real-world conditions of such a service.

Furthermore, patients were assessed only once during a TTYT consultation, with no subsequent sessions with different clinicians, contributing to variability among patients and clinicians. This variability complicates direct comparisons in MRP identification and intervention, potentially weakening the analysis of intervention types across different clinicians. In this pragmatic retrospective analysis of real-world data, despite efforts to minimise dependent variables by prioritising clinician types over individual patient records and analysing only the primary MRP for each encounter, variations across encounters cannot be accounted for as well as in a randomised study design.

Moreover, in clinical practice, triaging processes are used to assign patients to the most appropriate clinician for optimal patient care [36]. The triaging process to different clinicians in TTYT may have introduced selection bias if pharmacists were selectively assigned patients with more medication changes during admission, potentially skewing results on pharmacist detection of MRPs during consultations. Finally, resource constraints limited the uniform collection of certain manual data (e.g., a-CCI scores, medication histories). The low Nagelkerke R^2^ value (< 0.05) for those independent variables suggested a limited fit of the model with the predictors, emphasising the necessity for further data to effectively identify predictors for patients using the TTYT service, which could have strengthened regression models and enhanced understanding of MRP risk factors in the study cohort.

As pharmacists resolved MRPs similarly to other team members and exhibited higher odds of MRP detection, further investigation into the cost-effectiveness of integrating pharmacists into similar services is warranted. These findings may bolster efforts to secure funding for future development of pharmacist services.

The study identified various types of MRPs and their interventions despite the current discharge process. Local dissemination of these findings prompted a review and update of discharge processes to minimise MRPs during care transitions. Other organisations may benefit from a similar improvement process. Moreover, investigating potential variations in MRP identification among clinicians attending the same patients is crucial for ensuring consistent, high-quality care for patients at risk of MRPs. Such inquiry could reveal clinical practice discrepancies and facilitate the development of standardised MRP detection tools and management practices.

## Conclusion

This study demonstrates no differences in intervention types to resolve MRPs between pharmacists and non-pharmacist clinicians in an immediate post-discharge TFU service for general medical patients. Additionally, the types of MRPs identified by these clinicians were also similar. Despite this alignment, the odds of a pharmacist identifying an MRP was more than double that of non-pharmacists' clinicians. These findings underscore pharmacists' comparable proficiency in identifying and resolving MRPs, highlighting their crucial role in transitional care services to increase patient safety.

## Supplementary Information

Below is the link to the electronic supplementary material.

Supplementary file1 (DOCX 169 KB)

## Data Availability

The datasets generated during and/or analysed during the current study are available from the corresponding author on reasonable request. All data supporting the findings of this study are available within the paper and its Supplementary Information.
