# Supplementary material for: Evaluation of clinical interventions made by pharmacists, doctors and nurses in a hospital post-discharge telephone service: a retrospective cohort study
Source: Int J Clin Pharm. 2026 Sep 2;48(6):2289–98. doi: 10.1007/s11096-026-02207-y (PMC13731706; doi:10.1007/s11096-026-02207-y)
Supplement: Supplementary file 1 [file 11096_2026_2207_MOESM1_ESM.docx]

**Supplementary Documents**

Supplementary Information (SI-1)- Table: Medication-related problems (MRPs) classification using NESA tool [20,21]

| **Medication-related problem category** | **Triggering subsets to be classified under the relevant MRPs category** |
| --- | --- |
| Necessity | Unnecessary Drug |
|  | Drug therapy required (new/additional) |
| Effectiveness | Ineffective drug: incorrect drug or drug product |
|  | Dose too low: correct drug but wrong dose |
| Safety | Dose too high – toxicity |
|  | Drug interaction |
|  | Adverse drug reaction |
| Adherence | Non-adherence: not taking enough drug |
|  | Over-adherence: taking too much drug |

Supplementary Information (SI-2) – Table: Illustrative Examples of medication related problems identified and interventions utilised to resolve.

| **Medication related problem category** | **Example of extracted data** | **Interventions** |
| --- | --- | --- |
| Necessity, N | Patient had wrist fracture and was told to take paracetamol and ibuprofen on discharge, but pain is still limiting hand movement despite having both paracetamol and ibuprofen. | Drug-level intervention: Oxycodone IR 5mg and docusate + sennosides tablets were additionally prescribed to help with pain management and prevention of opioids-induced constipation. |
|  | Patient’s vitamin D level is 184 (add units) and currently still taking 25micrograms of Vitamin D daily. | Drug-level intervention: Ceased Vitamin D tablets. |
| Effectiveness, E | Patient was still constipated despite having 1 sachet daily of Movicol (polyethylene glycol). | Drug-level intervention: Increased Movicol (polyethylene glycol) to 2 sachets twice daily. |
|  | Patient only lost 0.1kg despite on 1.5L fluid restriction and stated that the lower legs look a bit swollen. Usually on furosemide 40mg daily. | Drug-level intervention: Advised to increase furosemide to 40mg twice daily (morning + midday) and records daily weight. |
| Safety, S | Patient is experiencing drowsiness from current dose of Buprenorphine patch. | Other intervention: Liaised with GP to develop weaning plan for the buprenorphine patch. |
|  | Patient has intermittent nausea +/- vomiting post metformin commencement. | Drug-level intervention: Advised to cease metformin and monitor response. |
|  | Patient was taking Rivaroxaban and Paxlovid (Ritonavir/Nirmatrelvir) together. | Drug-level intervention: Informed patient that both medications can increase risk of bleeding due to drug-interaction between Paxlovid and Rivaroxaban. Advised patient to withhold rivaroxaban until 3 days post-completion of Paxlovid. |
| Adherence, A | Patient unaware that Perindopril and Atorvastatin were ceased. | Patient-level intervention: Went through discharge medication list and informed patient not to take atorvastatin and perindopril. |
|  | Patient was unsure how to use Clotrimazole cream both internally and externally. | Patient-level Intervention: Educated patient on applicator use for internal cream insertion. |
|  | Patient has difficulty using Symbicort (Budesonide/Formeterol)) Turbuhaler, and hence not using it at home. Familiar with Ventolin (salbutamol device), has been using Ventolin only. | Drug-level intervention and Patient-level intervention: Changed Symbicort Turbuhaler to Rapihaler (metered-dose inhaler) as it has similar method of using and patient can use it with spacer to help with dexterity. Counselled on the importance of using a preventer and the technique to use inhalers correctly. |
|  | Patient’s Daughter was unaware patient has to complete Molnupiravir course. | Patient-level intervention: Recommended to continue taking Molnupiravir until course completion. |

Supplementary Information (SI-3) – Table: Top 10 medications involved in medication related problems identified.

| **Medication** | **Number (%)**  **N=805** |
| --- | --- |
| Furosemide | 57 (7.1%) |
| Apixaban | 31 (3.8%) |
| Paracetamol | 30 (3.7%) |
| Budesonide (inhaled) | 25 (3.1%) |
| Oxycodone | 19 (2.4%) |
| Aspirin | 19 (2.4%) |
| Docusate/Sennosides | 19 (2.4%) |
| Prednisolone | 17 (2.1%) |
| Metoprolol | 15 (1.9%) |
| Amlodipine | 13 (1.6%) |

Supplementary Information (SI-4) – Figure: Distribution of different types of interventions utilised to address each medication related problem (MRP) type identified by different clinicians. *MRPs Medication-related problems, N Necessity, E Effectiveness, S Safety, A Adherence.*

Interventions utilised to address categorised MRPs

| Categorised MRPs utilised by different Clinicians | Prescriber Level | Patient Level | Drug Level | Other intervention (liaison) |
| --- | --- | --- | --- | --- |
| Pharmacist N | 3.8% | 38.5% | 46.2% | 11.5% |
| Pharmacist E | 27.3% | 27.3% | 45.5% | 0% |
| Pharmacist S | 3.8% | 80.8% | 11.5% | 3.8% |
| Pharmacist A | 4.2% | 81.2% | 6.1% | 8.5% |
| Nurse N | 0% | 83.3% | 16.7% | 0% |
| Nurse E | 14.3% | 57.1% | 14.3% | 14.3% |
| Nurse S | 5.9% | 64.7% | 29.4% | 0% |
| Nurse A | 0.9% | 88.7% | 5.7% | 4.7% |
| Doctor N | 3.7% | 40.7% | 51.9% | 3.7% |
| Doctor E | 12.5% | 50% | 37.5% | 0% |
| Doctor S | 0% | 38.1% | 52.4% | 9.5% |
| Doctor A | 8.5% | 74.5% | 12.3% | 4.7% |

Supplementary Information (SI-5) – Figure: Distribution of medications involved in the Medication-related problems identified within the top 5 medication categories.

|  |  |
| --- | --- |
| A:Alimentary Tract and metabolism A12A | 2 |
| A:Alimentary Tract and metabolism A02B | 15 |
| A:Alimentary Tract and metabolism A03F | 4 |
| A:Alimentary Tract and metabolism A04A | 3 |
| A:Alimentary Tract and metabolism A06A | 43 |
| A:Alimentary Tract and metabolism A07A | 5 |
| A:Alimentary Tract and metabolism A10A | 18 |
| A:Alimentary Tract and metabolism A10B | 34 |
| A:Alimentary Tract and metabolism A11C | 16 |
| A:Alimentary Tract and metabolism A12B | 6 |
| A:Alimentary Tract and metabolism A12C | 3 |
| B: Blood and blood forming organs. B01A | 71 |
| B: Blood and blood forming organs. B02B | 1 |
| B: Blood and blood forming organs. B03A | 3 |
| B: Blood and blood forming organs. B03B | 1 |
| B: Blood and blood forming organs. B03X | 1 |
| B: Blood and blood forming organs. B05C | 1 |
| B: Blood and blood forming organs. B05X | 5 |
| C: Cardiovascular system C01A | 6 |
| C: Cardiovascular system C01B | 6 |
| C: Cardiovascular system C01C | 3 |
| C: Cardiovascular system C01D | 11 |
| C: Cardiovascular system C02A | 8 |
| C: Cardiovascular system C02C | 1 |
| C: Cardiovascular system C03A | 2 |
| C: Cardiovascular system C03B | 4 |
| C: Cardiovascular system C03C | 58 |
| C: Cardiovascular system C03D | 12 |
| C: Cardiovascular system C07A | 35 |
| C: Cardiovascular system C08C | 20 |
| C: Cardiovascular system C08D | 2 |
| C: Cardiovascular system C09A | 14 |
| C: Cardiovascular system C09B | 4 |
| C: Cardiovascular system C09C | 23 |
| C: Cardiovascular system C09D | 13 |
| C: Cardiovascular system C10A | 32 |
| N: Nervous system N02A | 45 |
| N: Nervous system N02B | 39 |
| N: Nervous system N02C | 1 |
| N: Nervous system N03A | 4 |
| N: Nervous system N04B | 1 |
| N: Nervous system N05A | 13 |
| N: Nervous system N05C | 7 |
| N: Nervous system N06A | 9 |
| N: Nervous system N07B | 1 |
| N: Nervous system N07C | 2 |
| J: Anti-infective for systemic use. J01A | 11 |
| J: Anti-infective for systemic use. J01C | 21 |
| J: Anti-infective for systemic use. J01D | 10 |
| J: Anti-infective for systemic use. J01E | 5 |
| J: Anti-infective for systemic use. J01F | 3 |
| J: Anti-infective for systemic use. J01M | 3 |
| J: Anti-infective for systemic use. J01X | 5 |
| J: Anti-infective for systemic use. J04A | 1 |
| J: Anti-infective for systemic use. J05A | 17 |

**Appendix 1: STROBE checklist for Cohort study**.
